# Acceleration of wound healing by topical application of gel formulation of *Barringtonia racemosa* (L.) Spreng kernel extract

**DOI:** 10.12688/f1000research.104602.2

**Published:** 2022-03-16

**Authors:** Nur A. Sitohang, Effendy D. L. Putra, Hajjul Kamil, Musri Musman

**Affiliations:** 1 Graduate School of Mathematics and Applied Science, Universitas Syiah Kuala, Banda Aceh, 23111, Indonesia; 2 Faculty of Nursing, Universitas Sumatera Utara, Medan, 20222, Indonesia; 3 Faculty of Pharmacy, Universitas Sumatera Utara, Medan, 20222, Indonesia; 4 Faculty of Nursing, Universitas Syiah Kuala, Banda Aceh, 23111, Indonesia; 5 Faculty of Education and Teachers’ Training, Universitas Syiah Kuala, Banda Aceh, 23111, Indonesia

**Keywords:** Barringtonia, Gel, Lecythidaceae, Phytomedicine, Wound healing

## Abstract

**Background**: Phytomedicines are gaining a spotlight in wound management, where much research has suggested the wound healing potential of *Barringtonia racemosa*. The objective of this study was to investigate the effectiveness of *B. racemosa* kernel extract in accelerating wound healing process in animal models.

**Methods:** *B. racemosa* kernel was extracted using ethanol:water (7:3) solvent and was then used as a bioactive ingredient in a Carbopol 940-based gel formulation in four different concentrations (1, 3, 5 and 7 ppm). A 3 cm diameter wound was made in the dorsal area of *Rattus norvegicus* rat and wound healing process was assessed up to 12 days using DESIGN (Depth, Exudate, Size of Inflammation/Infection, Granulation tissue, and Necrotic tissue) scoring system.

**Results:** Our data suggested that the DESIGN scores were significantly different among concentration groups after the 3 ^rd^day onward suggesting *B. racemosa* extract accelerated the wound healing process. Rats treated with gel formulation containing 7 ppm of *B. racemosa* kernel extract had faster wound healing than that treated with topical Metcovazin. On day 6, macroscopic observation on 7 ppm group revealed that the wound had persistent redness, lesion area of < 3 cm ^2^, and 80% healthy granulation, where presence of exudate and redness were not observable.

**Conclusion:** *B. racemosa* kernel extract was effective in accelerating wound healing on rats. Further study is warranted to purify the bioactive component and the action mechanism in wound healing process.

## Introduction

Wounds may occur on skin because of external or internal stimuli including that of physical, chemical, electrical, or thermal. Injured skin loses its function, causing it to be unable to protect the body from the entry of pathogenic organisms. ^1^^,^^2^ Wound healing could be initiated rapidly owing to body’s hemostasis system. Overlapping, yet distinctive, stages of wound healing are inflammation, proliferation, and remodeling. ^3^ Of which, inflammation is the most crucial phase that occurs to protect the wound from bacterial infection and promoting tissue repair. ^3^^,^^4^ Therefore, many researches focused on improving wound management in particular during the inflammation stage. ^5^ Several traditional medicines have been used in wound dressing to accelerate the healing process, including those consisting of plant extracts. ^6^^,^^7^

Emerging research has tried to provide evidence-based phytomedicines as a candidate to treat diseases including as substitute or addendum in wound management. ^8^^–^^10^ There have been many examples of plant-based traditional medicines possessing wound healing properties. ^9^^,^^10^ This could be attributed to the high content of bioactive compounds in plant extract such as saponins, flavonoids, terpenoids, and tannins. ^6^^,^^11^ *Barringtonia racemosa* (L.) Spreng is among commonly used plants in ethnomedicine which has been reported to contain diterpenes, triterpenoids, flavonoids, steroids and saponins. ^12^ A previous phytochemical screening reported that extracts from kernels, barks, and stems of *B. racemos*a contained a rich amount of saponins. ^13^ Furthermore *B. racemos*a extract has been reported to be active against Gram-positive and -negative bacteria such as *Staphylococcus aureus*, *Staphylococcus epidermidis*, *Eschericia coli*, *Shigella dysentriae*, *Vibrio cholerae* and Proteus sp. ^14^ Its potential as a wound healing therapy was further corroborated by the studies reporting antinociceptive (analgesic), antioxidant, anti-inflammatory, and anti-fungal properties of *B. racemosa* extracts. ^14^^,^^15^

Current research has not reported *B. racemosa* extract's potential in wound healing. Indeed, there are studies that have employed plant extracts from the same family (Lecythidaceae) ^16^ or the same genus (Barringtonia). ^17^ Although the phytochemical constituents might be similar across plants in the same family or genus, differences still expected between species. ^18^ Currently, there are no studies reporting *in-vivo* investigation of wound healing potential of *B. racemosa* extract. Hence, this study sought to investigate the wound healing effect of *B. racemosa* extract in an animal model. Several animals have been used to study the wound healing processes including to evaluate efficacy of different treatment modalities. ^19^ However, rats have been widely used in the study of skin wound healing because of their availability, low cost, and small size. ^20^ Studies have used rats as an animal model to evaluate the effect of several natural extracts on would healing. ^21^^–^^24^ The kernel was used since most of secondary metabolites stored in the kernel act as a surviving mechanism of the plant. ^25^ After the extract was obtained, the gel formulation was made with beneficial characteristics for wound healing including high content of water, flexibility and biocompability. ^26^ The prepared formulation was then used to treat the wound in animal models.

## Methods

### Ethics

The protocol of this study was approved by the Ethics Committee of the Faculty of Mathematics and Natural Sciences, Universitas Sumatera Utara, Indonesia (#0700/KEPH-FMIPA/2019). All efforts were made to ameliorate the suffering of the rats; criteria (described below) were established to ensure that any subjects could be excluded if need be. After the experiment, all animals were returned to the breeding center of Pharmacology Laboratory at Faculty of Pharmacology, Universitas Sumatra Utara.

### Materials

Materials used in this research were pharmaceutical grade chemicals including ethanol 96%, Carbopol 940, triethylamine, propylene glycol, and methyl paraben (Merck, Selangor, Malaysia). Ripe fruits of *B. racemosa* (L.) Spreng were collected from Desa Gampong Pulo, Bireuen Regency, Aceh, Indonesia.

### Study design

The study was conducted in the Pharmacy Laboratory of Faculty of Pharmacy, Universitas Sumatera Utara, Medan, Indonesia. The wound healing effect of gel formulation of *B. racemosa* extract was assessed in rat models ( *Rattus norvegicus*) and the wound healing process was assessed using DESIGN scoring system (Depth, Exudate, Size of Inflammation/Infection, Granulation tissue, and Necrotic tissue).

### *B. racemosa* kernel extraction

The white‐colored kernels of *B. racemosa* were taken from the fruits, washed repeatedly using water, and sun-dried. Dried kernels were cut into pieces with 1 cm in diameter, oven-dried at 40°C, and crushed into powder using a DF-15 grinder (Cgoldenwall) before sifted through a 60-mesh sieve. Maceration was then conducted on the *B. racemosa* kernel powder in a ethanol:water (7:3) solvent for 24 hours. This process was repeated until the solvent turned colorless. The produced filtrate was concentrated using a Hei-VAP Expert rotary evaporator (Heidolph Instruments GmbH & CO. KG, Schwabach, Germany) for 30 minutes.

### Gel preparation

**Figure 1.**
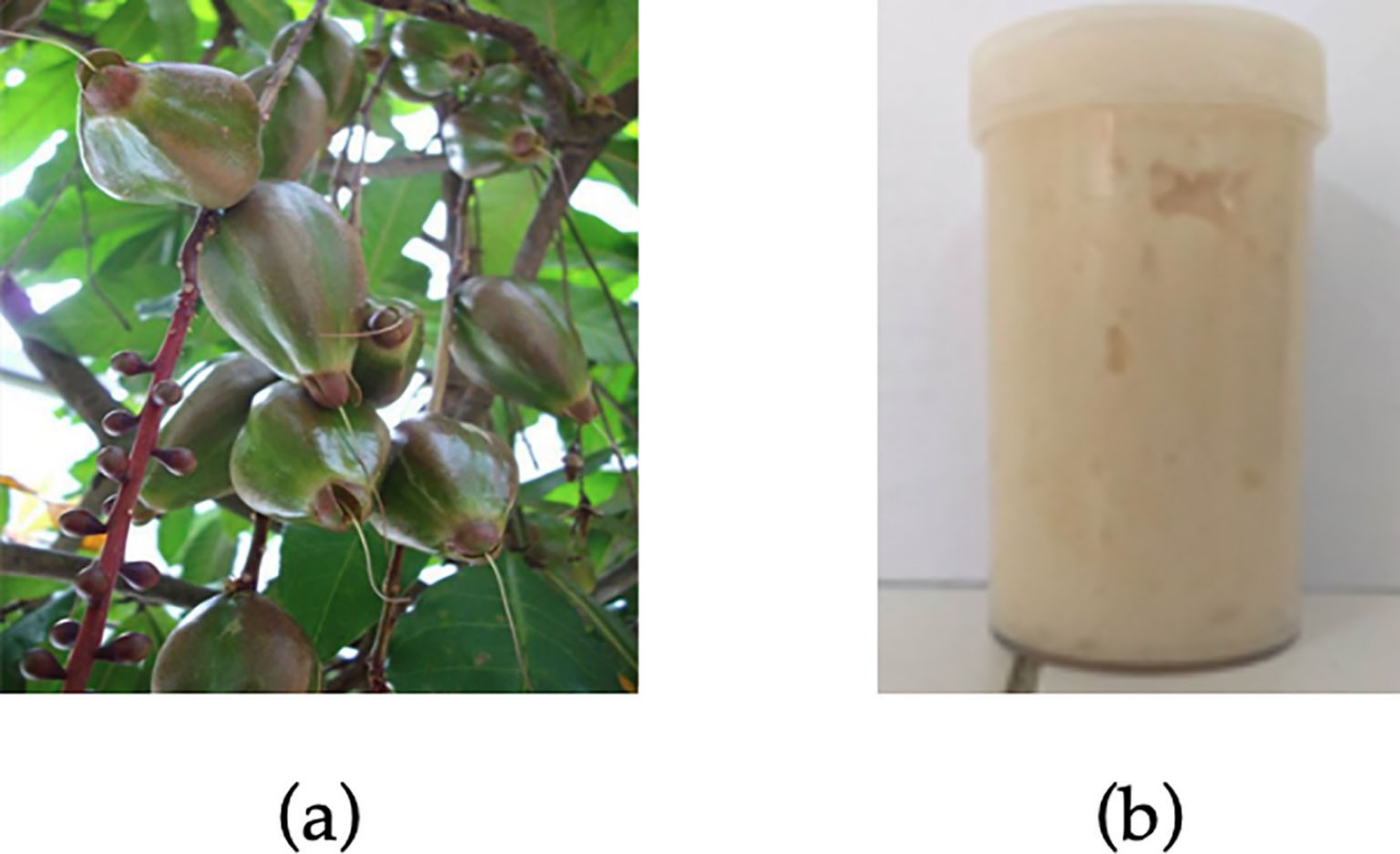
(a) *Barringtonia racemosa* (L.) Spreng and (b) its ethanolic extract gel.

Gel formulation was prepared using Carbopol 940, triethylamine, propylene glycol, methyl paraben, and distilled water following our previous report. ^27^ Briefly, 0.2 g methyl paraben (0.2% w/w) was dissolved in 100 mL pre-heated distilled water (70°C), and then added with 2 g Carbopol 940 to form the gel. The mixture was stirred by hand until foam and gel were formed. Subsequently, 6 g propylene glycol (6% w/w) and 2 g triethylamine (2% w/w) were added into the gel solution. Lastly, *B. racemosa* kernel was added to gel formulation with a concentration of 1, 3, 5, and 7 ppm (mg/kg). The *B. racemosa* fruits and prepared gel formulation are presented in Figure 1.

### Animal model

A total of 24 male rats ( *Rattus norvegicus*) Wistar strain with average body weight (200-300 g) and age of 2-3 months old were acclimated and fed *ad libitum* with standardized feed (RatBio ^®^). This animal model has been used to assess the effects of some drugs on wound healing process. ^28^^,^^29^ All conditions (i.e., temperature, humidity and lighting) followed the guideline from Institute for Laboratory Animal Research, USA. ^30^ The acclimatization process was conducted for a week prior to the study under laboratory conditions with 60% humidity, temperature 23±1°C, and 12h light-dark cycle. Afterward, the 24 rats were randomly divided into six groups of four; a negative control, a positive control, and four treatment groups based on the Federer formula. ^31^ The mathematical expression of Federer formula is shown below:

$$\left(t-1\right)\left(n-1\right)>15$$ (1)

Where t and n are number of group and number of subjects in a group, respectively. As for the randomization, it was performed on an online web-based randomizer tool ( https://www.randomizer.org/). 

The same laboratory conditions of acclimatization (i.e., 60% humidity, temperature 23±1°C, and 12h light-dark cycle) were used during the actual study.

### *B. racemosa* gel application

Dorsal area of the rats was shaved with a diameter of 3 cm around the incision area. Prior to incision, the skin was disinfected using rubbing alcohol 70% and the rats were anesthetized using intramuscular injection of ketamine and xylazine (75 mg/kg body weight). A skin incision was made with a wound length of 2 cm and a depth of 0.2 cm to the dermis, as suggested previously. ^16^^,^^17^ Each treatment group received 100 mg gel formulation with *B. racemosa* kernel extract of 1 ppm, 3 ppm, 5 ppm, and 7 ppm each. The study was controlled by positive and negative control groups. Negative control was not administered any treatment, and the positive control was smeared with 100 mg of 25 g Metcovazin cream consisting of zinc oxide and chitosan.

### Assessment of healing process

The wound healing process was observed once daily for 12 days and recorded based on DESIGN scores. Detailed components assessed in DESIGN scoring system could be seen as follows: depth (0-5), exudate (0-6), wound area (0-15), infection (0-9), granulation (0-6), necrotic (0-6), and pocket (0-24). ^32^ For each animal, the DESIGN scores were summed to give a single score (ranging from 0 to 71); the lower the score better the healing process. The data were presented descriptively in mean ± standard deviation. In each observation, the body weight of the animals and the behavior were observed. Rapid weight loss of 15% and abnormal behaviors such as apathy or increased aggression were set as humane endpoints to exclude the animal from the study. After the study completion, all animals were returned to the breeding center of Pharmacology Laboratory at Faculty of Pharmacology, Universitas Sumatra Utara.

### Statistical analysis

Kolmogorov-Smirnov test was employed to assess the normality of the data distribution in each group. Normally distributed data were then tested using Kruskal-Wallis test to compare DESIGN scores obtained from different groups on the same observation day. Meanwhile, Mann-Whitney test was used to compare data from two different groups collected on the same day. All analyses were conducted on GraphPad Prism 9.2.0 software (GraphPad Software, San Diego, CA, USA) or free alternatives such as JASP or SOFA Statistics could be used.

## Results

### Wound healing effect of *B. racemosa* extract gel

Periodical changes of DESIGN scores observed in the rats are presented in Table 1. Negative control had a gradual reduction of DESIGN score but still persisted even after 12 days. Similar results were also observed in the treatment group with *B. racemosa* 1 ppm extract concentration. When the extract concentration in gel formulation was increased to 3 or 5 ppm, the wound resolved after 12 days of observation. The results were similar to that obtained in positive control which received commercial wound healing cream – Metcovazin. Interestingly, *B. racemosa* extract with a concentration of 7 ppm could perform better wound healing on the rat models; this condition yielded 0 DESIGN score on the 11 ^th^ day. DESIGN scores, observed from day 3 to day 12, between studied groups had statistical significance ( *p*<0.001) suggesting the extract could effectively accelerate the wound healing process. On day 11, the DESIGN scores were significantly different between 7 ppm group and positive control group ( *p*=0.0286, Mann-Whitney test) suggesting that *B. racemosa* extract with a concentration of 7 ppm in gel formulation had at least equal wound healing properties equal to that of Metcovazin (in vivo).

**Table 1.** DESIGN score recorded for 12 days of *B. racemosa* extract gel treatment.

Group	DESIGN mean score ± SD					
	Day 0	Day 3	Day 6	Day 9	Day 11	Day 12
Negative control	15 ± 0.00	13 ± 0.00	11.5 ± 1.00	8 ± 2.00	3.5 ± 1.73	2.25 ± 1.89
1 ppm	15 ± 0.00	12 ± 0.00	10 ± 0.00	9 ± 0.00	5 ± 0.00	2 ± 0.00
3 ppm	15 ± 0.00	12 ± 0.00	10 ± 0.00	5 ± 0.00	2 ± 0.00	0 ± 0.00
5 ppm	15 ± 0.00	12 ± 0.00	10 ± 0.00	5 ± 0.00	2 ± 0.00	0 ± 0.00
7 ppm	15 ± 0.00	10 ± 0.00	9 ± 0.00	2 ± 0.00	0 ± 0.00	0 ± 0.00
Positive control	15 ± 0.00	10 ± 0.00	9 ± 0.00	5 ± 0.00	1.5 ± 0.58	0 ± 0.00
*p-*value ^*^	NA	0.0003 ^*^	0.0006 ^*^	0.0008 ^*^	0.0013 ^*^	0.0005 ^*^

Positive control group received Metcovazin cream. NA: not applicable.

^*^ Significant at *p*<0.001 based on Kruskal-Wallis test.

### Macroscopic observation of treatment

**Figure 2.**
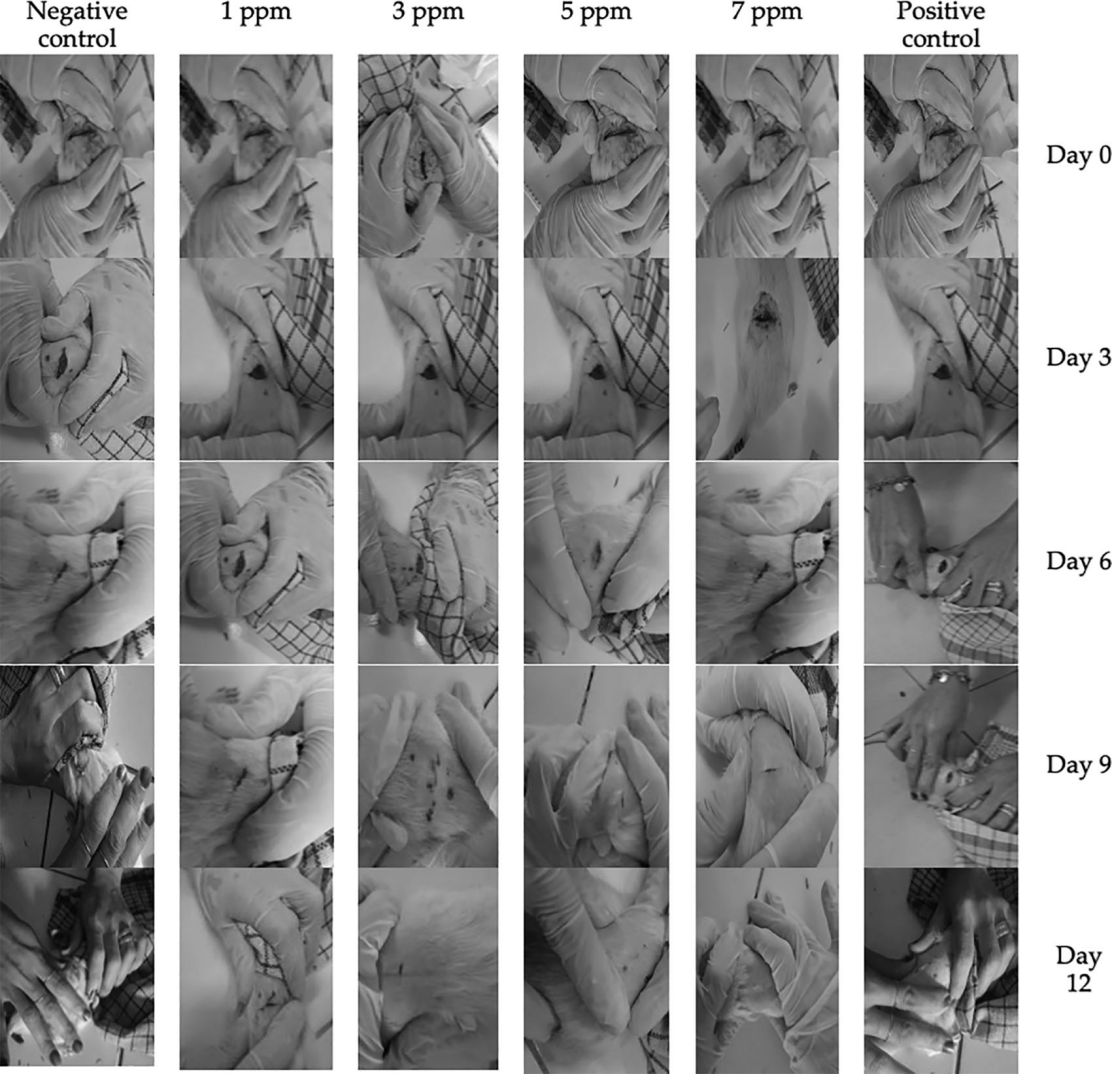
Wound healing process in rats after the treatment using *B. racemosa* extract gel. Positive control group received Metcovazin cream.

During the first 3 days of treatment, all groups have entered inflammatory phase characterized by the presence of fluids, lesion area of <4 cm ^2^, redness, and swelling ( Figure 2). Thereafter, rats underwent proliferation phase indicated by light exudate, lesion area of <3 cm ^2^, redness and healthy granulation of <10%. In general, the proliferation phase could be observed lasted until the 9 ^th^ day. Faster wound healing process could be found in a group treated with 7 ppm *B. racemosa* extract, where on the 6 ^th^ day, the wound had persistent redness, less than 3 cm ^2^ lesion area, and 80% healthy granulation without the presence of exudate and redness ( Figure 2).

After 12 days of observation, most of the wounds had reached remodeling phase. There were two rats in negative control group that experienced remodeling phase accompanied with persistent redness and healthy granulation (>90%), where no exudate, wounded area, and signs of inflammation ( Figure 2). However, the two other rats in the negative control group still had 4 cm ^2^ lesion. In all animals within 1 ppm of *B. racemosa* extract, the wound persisted with an area of less than 4 cm ^2^ with no sign of infection and 95% of the wound had healthy granulation. When the concentration was increased to 3 or 5 ppm, the wounds were completely closed with granulation reaching 90% or more. Similar results were also obtained in the positive group. The fastest wound healing was found in treatment group receiving gel containing 7 ppm *B. racemosa* extract, where wound area and granulation were no longer observable ( Figure 2).

## Discussion

Wound healing is a complex dynamic process in the restoration of tissue anatomy function after injury. ^3^ It is necessary to maintain the condition that is ideal for cell repair and regeneration during the wound healing. Collagen production, angiogenesis, and granulation have important role in healing process. For its optimization, effective therapies using wound healing agents are required. ^5^^,^^7^^,^^33^ Of which, hydrogels have been proposed as strong drug delivery media for acute open wound management due to their high content of water, biocompatibility, and flexibility. ^5^^,^^26^

In this present study, we used gel formulation of *B. racemosa* extract for open wound treatment on rat models. Our data suggest the extract with a concentration of 7 ppm could accelerate the wound healing progress and is not inferior to Metcovazin. This suggests wound healing properties of phytocompounds contained in the *B. racemosa* extract. Previously, a study found that aqueous extract of *B. racemosa* contains diterpenes, triterpenoids, flavonoids, steroids and saponins. ^12^ A study had successfully isolated two triterpenoids, barringtogenol and barringtogenic acid from the seeds of *B. racemosa* using ethanol 70%. ^34^ It was the only study who had identified the pure compounds of *B. racemosa* seeds, ^34^ where the biological activities of the compounds have not investigated yet. Saponins, in animal models, have been known to improve immune system, optimize blood sugar levels and reduce blood clots. ^35^ Saponins also contribute to induce collagen production in skin fibroblasts via phosphorylation of Smad2 protein. ^36^ Furthermore, antioxidant and antibacterial properties of saponins could assist faster wound healing. ^37^ Other than saponins, flavonoids have been reported to increase collagen secretion and consequently induce tissue granulation. ^13^ Studies also prove that flavonoids have anti-inflammatory properties. ^38^^,^^39^

Tannins also play a role in the wound healing process since they could act as astringents. ^40^ Astringent causes reduced mucosal permeability, leading to stronger bonds between the mucosa which inhibits the entrance of microorganisms and irritant chemicals. ^37^^,^^41^ Furthermore, tannins are reported to be capable of inhibiting hypersecretion of mucosal fluid and neutralizing inflammatory proteins. ^42^ Tannin compounds promote antibacterial activities by hampering the permeability the bacterial cell wall. ^43^ Wound healing properties are also possessed by terpenoids contained in *B. racemosa* extract including antimicrobial, antifungal, antiviral, antiparasitic, antihyperglycemic, antiallergenic, anti-inflammatory, antispasmodic, immuno-modulatory, and chemotherapeutic properties. ^14^^,^^15^

Wound healing activities of plants from family Lecythidaceae have been reported by previous studies. ^16^^,^^17^ Wound contraction of 125-140% was obtained after the treatment using topical treatment of *Napoleona vogelii* leaf extract. ^16^ Topical administration of *Barringtonia acutangula* fruit as much as 20% w/w yielded 86.3% recovery after 14 days of treatment. ^17^ Those stated results could not be compared to our study because of the use of different assessment method. Nonetheless, we argued that DESIGN scoring could provide more comprehensive assessment as it includes observations on wound depth, exudate secretion, wound size, infection, granulation, and necrotic tissue. Our collective findings suggest that B. *racemosa* 7 ppm in gel formulation could be used in acute wound management via topical administration. In gel formulation, the wound healing drug has an advantage of being flexible and capable of controlling the humidity around the wound area. However, as the disadvantage, patients might feel discomfort of using gel-based drug. As a limitation, this study was unable to identify the compound responsible for the healing properties. Therefore, further studies were required to figure out the bioactive compounds contained in the *B. racemosa* extract.

## Conclusions

*B. racemosa* extract in gel formulation was effective for wound treatments in rat models. A formulation with *B. racemosa* extract concentration as low as 3 ppm could exhibit the recovery rate similar to that obtained from those treated with Metcovazin. In animals treated with gel formulation of 7 ppm, the DESIGN score reached 0 sooner than that treated with Metcovazin. Taken together, gel formulation of *B. racemosa* extract is potentially be used to treat wound since it accelerates the wound healing process. In addition, gel as a delivery medium was found suitable for the *B. racemosa* extract. In future studies, it is interesting to investigate *B. racemosa* extract for wound treatment with more complex condition, such as diabetic wound, either by using gel as the delivery medium or others.

## Data availability

### Underlying data

figshare: Acceleration of wound healing by topical application of gel formulation of Barringtonia racemosa (L.) Spreng kernel extract. https://doi.org/10.6084/m9.figshare.17256023.v6. ^44^

This project contains the following file:

- Master.xlsx (DESIGN scores from 0 – 15 for all groups)

Data are available under the terms of the Creative Commons Attribution 4.0 International license (CC-BY 4.0).

## Reporting guidelines

ARRIVE Essential 10 checklist for ‘Acceleration of wound healing by topical application of gel formulation of *Barringtonia racemosa* (L.) Spreng kernel extract’. https://doi.org/10.6084/m9.figshare.18866303. ^45^

Data are available under the terms of the Creative Commons Attribution 4.0 International license (CC-BY 4.0).
