# Supplementary material for: Acceleration of wound healing by topical application of gel formulation of Barringtonia racemosa (L.) Spreng kernel extract
Source: F1000Res. 2022 Mar 16;11:191. Originally published 2022 Feb 15. [Version 2] doi: 10.12688/f1000research.104602.2 (PMC8933646; doi:10.12688/f1000research.104602.2)
Supplement: Peer review history

# Peer review: Reviewer response for version 2

**DOI:** 10.5256/f1000research.122388.r127795

**Published:** 2022-03-18

**Authors:** Trina Ekawati Tallei

**Affiliations:** 1 Department of Biology, Faculty of Mathematics and Natural Sciences, Sam Ratulangi University, Manado, Indonesia

After carefully reading the revised article, I have come to the conclusion that the revised results are acceptable and that the indexing of the article is recommended.

Is the work clearly and accurately presented and does it cite the current literature?

Yes

If applicable, is the statistical analysis and its interpretation appropriate?

I cannot comment. A qualified statistician is required.

Are all the source data underlying the results available to ensure full reproducibility?

Partly

Is the study design appropriate and is the work technically sound?

Partly

Are the conclusions drawn adequately supported by the results?

Yes

Are sufficient details of methods and analysis provided to allow replication by others?

Yes

Reviewer Expertise:

Drug discovery and pharmacoinformatics.

I confirm that I have read this submission and believe that I have an appropriate level of expertise to confirm that it is of an acceptable scientific standard.

# Peer review: Reviewer response for version 2

**DOI:** 10.5256/f1000research.122388.r127794

**Published:** 2022-03-17

**Authors:** Talha Bin Emran

**Affiliations:** 1 Department of Pharmacy, BGC Trust University Bangladesh, Chittagong, Bangladesh

The authors did a great job. The revised manuscript can be accepted for indexing.

 Congratulations!

Is the work clearly and accurately presented and does it cite the current literature?

Yes

If applicable, is the statistical analysis and its interpretation appropriate?

Yes

Are all the source data underlying the results available to ensure full reproducibility?

Yes

Is the study design appropriate and is the work technically sound?

Yes

Are the conclusions drawn adequately supported by the results?

Yes

Are sufficient details of methods and analysis provided to allow replication by others?

Yes

Reviewer Expertise:

Natural Product Chemistry, Immunology

I confirm that I have read this submission and believe that I have an appropriate level of expertise to confirm that it is of an acceptable scientific standard.

# Peer review: Reviewer response for version 1

**DOI:** 10.5256/f1000research.114838.r123769

**Published:** 2022-03-08

**Authors:** Trina Ekawati Tallei

**Affiliations:** 1 Department of Biology, Faculty of Mathematics and Natural Sciences, Sam Ratulangi University, Manado, Indonesia

I would like to comment on the study design. It has been previously published elsewhere but the authors did not cite it. ^1^

 The authors did not mention how many replications they made for each concentration (ppm).

 The discussion should be expanded including supposition of what compounds (not groups of compounds) that play a role in wound healing which is supported by the literature. The group of compounds alone is still too general. The author should also discuss the mechanism of the wound healing process and relate it to their findings.

 Authors should also discuss the use of DESIGN scoring, as compared to previous studies using the same method.

 Authors are advised to describe the clinical application of the findings, advantages, and disadvantages.

Is the work clearly and accurately presented and does it cite the current literature?

Yes

If applicable, is the statistical analysis and its interpretation appropriate?

I cannot comment. A qualified statistician is required.

Are all the source data underlying the results available to ensure full reproducibility?

Partly

Is the study design appropriate and is the work technically sound?

Partly

Are the conclusions drawn adequately supported by the results?

Yes

Are sufficient details of methods and analysis provided to allow replication by others?

Yes

Reviewer Expertise:

Drug discovery and pharmacoinformatics.

I confirm that I have read this submission and believe that I have an appropriate level of expertise to confirm that it is of an acceptable scientific standard, however I have significant reservations, as outlined above.

## References

[1] : Formulation and Evaluation of Gel Containing BarringtoniaRacemosa L.Spreng Kernel Extract for Topical Application. *Proceedings of the International Conference on Health Informatics and Medical Application Technology* .2020; Reference source

# Response

**Published:** 2022-03-12

**Authors:** Musri Musman

**Affiliations:** Universitas Syiah Kuala, Indonesia

1. The previous publication only reports on the preparation of the gel formulation and its characteristics. It has been cited in the Methods.

 2. The number of replications is based on the number of samples per group (4 rats = 4 replications for each concentration).

 3. As of now, there are only two compounds successfully isolated from the seed of *B. racemosa*; barringtogenol and barringtogenic acid. Hence, other than the two compounds, we could only provide general group of compounds.

 4. We have tried our best searching DESIGN scoring in *in vivo* study, but still could not find the comparable study.

 5. Yes, we agree that information about the clinical application, advantages, and disadvantages should be added. We have added such information at the end of the discussion.

# Peer review: Reviewer response for version 1

**DOI:** 10.5256/f1000research.114838.r123768

**Published:** 2022-03-02

**Authors:** Talha Bin Emran

**Affiliations:** 1 Department of Pharmacy, BGC Trust University Bangladesh, Chittagong, Bangladesh

Title: Acceleration of wound healing by topical application of gel formulation of *Barringtonia racemosa* (L.) Spreng kernel extract

**Minor comments:**

 Although the article has scientific rigor, several minor flows need to be improved before indexing.

1.  The abstract section is unsuitable—no focus point in the abstract section.
2. Aim of the study need to write as the last paragraph of the introduction.
3. DESIGN scoring system: Any references?
4. Statistical analysis: Need some details.  
5. On day 11, the DESIGN scores were significantly different between the 7 ppm group and positive control group (p=0.0286, Mann-Whitney test) suggesting that *B. racemosa* extract with a concentration of 7 ppm in gel formulation could assist better wound healing than Metcovazin. Is this enough to conclude?
6. The discussion is feeble. Please, include the data from other sources about related works.
7. Figures presentation are good.
8. Figure legends are self-explanatory.
9. The conclusion needs to address future perspectives.

Is the work clearly and accurately presented and does it cite the current literature?

Yes

If applicable, is the statistical analysis and its interpretation appropriate?

Yes

Are all the source data underlying the results available to ensure full reproducibility?

Yes

Is the study design appropriate and is the work technically sound?

Yes

Are the conclusions drawn adequately supported by the results?

Yes

Are sufficient details of methods and analysis provided to allow replication by others?

Yes

Reviewer Expertise:

Natural Product Chemistry, Immunology

I confirm that I have read this submission and believe that I have an appropriate level of expertise to confirm that it is of an acceptable scientific standard, however I have significant reservations, as outlined above.

# Response

**Published:** 2022-03-12

**Authors:** Musri Musman

**Affiliations:** Universitas Syiah Kuala, Indonesia

1. We have added more information, especially in the results part, to give readers clearer understanding on the study.

 2. We have added the objective of the study in the last paragraph of the introduction, where it aimed to investigate the wound healing effect of *B. racemosa* extract in an animal model

 3. There is a reference for DESIGN score. (Horn *et al.* Adv Skin Wound Care. 2015; 28:560-72.) This reference has been cited in the text.

 4. The statistical analysis has been revised.

 5. We agree with the reviewer. However, the data are sufficient to conclude that at least *B. racemosa* extract 7 ppm has a similar wound healing property to that of Metcovazin (in vivo).

 6. The data have been compared with those from family Lecythidaceae. The data could not be compared quantitatively because other studies did not use the DESIGN score. This explanation has been added in the last paragraph of the discussion.

 7. We have revised the conclusions by adding the probably future studies of employing *B. racemosa* extract in treating a diabetic wound
